# Supplementary material for: miR-128 regulates neuronal migration, outgrowth and intrinsic excitability via the intellectual disability gene Phf6
Source: eLife. 2015 Jan 3;4:e04263. doi: 10.7554/eLife.04263 (PMC4337614; doi:10.7554/eLife.04263)
Supplement: Supplementary file 1. — Contains Tables of LNA probe sequences, primers used in reporter, and expression plasmid cloning as well as qRT-PCR analysis. DOI: http://dx.doi.org/10.7554/eLife.04263.021 [file elife04263s001.docx]

Supplemental Experimental Procedures

Table 1 LNA probe sequences

| LNA Probe | Sequence |
| --- | --- |
| hsa-miR-128 | AAAGAGACCGGTTCACTGTGA |
| miR-128-1 precursor | GTGAGAAATGTAAACCTCTCAG |
| miR-128-2 precursor | GAGACCTGCTACTCACTCTC |
| hsa-miR-124 | GGCATTCACCGCGTGCCTTA |
| mmu-Phf6 cds | TCATCTTCTTCATCTCGCTCAT |

Table 2 Sensor assay primer sequences

| Primer | Sequence |
| --- | --- |
| Reelin For | TGACTCGAGTGGCGAACAGAAAGCCCTT |
| Reelin Rev | GCGGCCGCTGCTGGTGTTGGCTCCACCT |
| Jip3 For | CATGTCTCGAGAGAGGGATGAAGCAGGGTTT |
| Jip3 rev | ACTTGGAATTCCGTCACACTCATGTGGGAAC |
| Pard6b For | CATGTCTCGAGCGCAGTTGTCTTCCCTTACC |
| Pard6b Rev | ACTTGGGATCCCAACAGCACAGCATCTGGTT |
| Srgap2 For | CATGTCTCGAGGGGCACCCCTTGGTCCTTTGG |
| Srgap2 Rev | ACTTGGAATTCAGGGAGGTGACGTACAGCGG |
| Nrp2 For | TGAGCTCACCCTCAGATGGTCTC |
| Nrp2 Rev | ACAAATCATATAAACACAGTCTGACAT |
| Gria 3 For | TGAAGCCTGGAATTACAGCCCAC |
| Gria 3 Rev | TTGACTACCGCCAAACTCCC |

Table 3 qRT-PCR Primers

| Primer | Sequence |
| --- | --- |
| PHF6 For | GTGAGCCCCACTGCATTTCT |
| PHF6 Rev | CGCTGTCTCGTAGACCCTTTT |
| GAPDH For | AGGTCGGTGTGAACGGATTTG |
| GADPH Rev | TGTAGACCATGTAGTTGAGGTCA |
| Oaz1 For | AGTCAGCGGGATCACAGTCTT |
| Oaz1 Rev | AGGACCCTGGTCTTGTCGTTA |
| Beta-Actin For | GGCTGTATTCCCCTCCATCG |
| Beta-Actin Rev | CCAGTTGGTAACAATGCCATGT |
| Szrd1 For | ACAGCGGGAGGCAGAGTATG |
| Szrd1 Rev | AGGACCCAACGGCTGTCTGA |

Table 4 PHF6 primers

| Primer | Sequence |
| --- | --- |
| PHF6 sensor fw | TTTTCTCGAGGCTGGTAGAACAGCGTTTTG |
| PHF6 sensor rev | TTTTGGATCCCCCCAGGAACAAAGAGGTC |
| PHF6 cDNA fw | AAGGTCTCGAGATCATGTCAAGCTCAATTGA |
| PHF6 cDNA rev | AAGGTGAATTCAAGTGGAAAAGAAGCACAGGA |

Table 5 miR-128 binding site (sponge)

| UCACAGGUUAAAAAAUCUUGA |
| --- |
| Mismatches to the miR-128 sequence in red |
| G-U base pairs with miR-128 are underlined |

Table 6 Antibodies

| Antibody | Company | Concentration | Use |
| --- | --- | --- | --- |
| Anti-RFP | Abcam ab62341 | 1:150 | IF |
| Anti-GFP | Abcam ab13970 | 1:500 | IF |
| Anti-Tbr2 | Chemicon AB15894 | 1:200 | IF |
| Anti-DCX | Abcam ab18723 | 1:200 | IF |
| Anti-Dig-AP | Roche 11093274910 | 1:500 | ISH |
| Anti-PHF6 | Novus Biological NB-100-68261 | HEK-293 1:4000  Neurons 1:1500 | WB |
| Anti-PHF6 | BETHYL A301-451A | 1:100 | IF |
| Anti-streptavidin-HRP | Perkin Elmer TSA KIT | 1:500 | ISH |
| Anti-Cux1 | Santa Cruz sc-13024 | 1:150 | IF |
| Anti-Ctip2 | Abcam ab18465 | 1:200 | IF |
